# Supplementary material for: Cooperative root graft networks benefit mangrove trees under stress
Source: Commun Biol. 2021 May 5;4:513. doi: 10.1038/s42003-021-02044-x (PMC8100114; doi:10.1038/s42003-021-02044-x)
Supplement: Supplementary file 1 — Supplementary Information [file 42003_2021_2044_MOESM1_ESM.pdf]

Cooperative root graft networks benefit mangrove trees under stress

Alejandra G. Vovides, Marie-Christin Wimmler, Falk Schrewe, Thorsten Balke, Martin Zwanzig, Cyril Piou, Etienne Delay, Jorge López-Portillo, Uta Berger

Supplementary Information Contents

Supplementary Table 1. ....SI-2
Supplementary Methods SI-3
Supplementary Fig. 1 ....SI-4
Supplementary Fig. 2 ....SI-5
Supplementary Table 2. ....SI-6
Supplementary Fig. 3 ....SI-7
Supplementary Fig. 4 ....SI-8
Supplementary Fig. 5 ....SI-9
Supplementary Table 3 .... SI-10
Supplementary Fig. 6 .... SI-11
Supplementary References.. SI-12

Supplementary Table 1. Attributes of each forest stand. Stand density marked with* indicates stand density was calculated including all species. Numbers followed by parentheses report means (± SE), N denotes absolute values.

Forest stand	sediment salinity	*Stand density (trees ha^{-1})	*A. germinans* trees (N)	*A. germinans* density (trees ha^{-1})	Total grafted trees (%)	Top-height trees (N)	Top-height grafted trees (N)	Top-height grafted trees (%)
1	39.70(1.8)	600	27	300	70.4	6	6	100
2	45.35(1.7)	522	28	311	64.3	6	5	83
3	41.60(1.4)	933	55	611	34.5	12	8	67
8	58.62(1.2)	478	43	478	55.8	9	7	78
9	45.07(2.7)	856	47	522	68.1	10	8	80
10	46.90(1.5)	422	37	411	51.4	8	7	88
12	56.17(0.8)	900	81	900	44.4	17	9	53
13	58.07(1.0)	644	58	644	63.8	12	8	67

Total grafted trees = 56.6(4) %

Top-height grafted trees = 76.9(5) %

Supplementary Methods - Computation of asymmetric neighbourhood

Competition for resources is considered a main limiting factor for plant growth[1], and because plants, unlike animals, are unable to change their physical location, their resource acquisition depends on their local environment, including resource availability, plant size and the distance and size of neighbours with whom they compete[2,3]. This has driven the development of position-dependent competition indices[2]. The larger and closer a given plant's neighbours are, the stronger the competition the plant experiences, but this competition pressure can be reduced by the target plant's size. For example, competition is thought to be symmetrical if within a given group of trees all the neighbours are located at similar distances and are of a similar size (i.e. all neighbours possess similar chances to access resources). The opposite occurs for plants with contrasting size distributions, where bigger plants have a disproportionately larger effect on competition and resource acquisition[1] over smaller neighbours. This concept has stimulated the development of several methods to measure competition and study its effect on forest community dynamics[2]. A commonly used competition index is that developed by Hegyi[4], defined as

$$CI_{ij} = \frac{\left(\frac{D_{130i}}{D_{130j}}\right)}{dist_{i-j}},$$

where CI_{ij} represents the competition index between a target $tree_i$ and a given $neighbour_j$ is the stem diameter (D_{130}) of $neighbour_j$, whereas $dist_{i-j}$ is the distance between the $tree_i$ and its $neighbour_j$. This means that the larger D_{130_i} is relative to D_{130_j}, the smaller CI_{ij} becomes, and the cumulative competition (CI) for a $tree_i$ (the sum of competition values computed for each neighbour of $tree_i$: $CI = \sum_j CI_{ij}$) decreases as the stem diameters of target trees become wider (Supplementary Figure 1a). Then, CI is a measure of competition autocorrelated to tree size; however, when assessing the effect of CI on tree allometry, statistical assumptions of independence between variables are violated, so no conclusions can be drawn as to the potential effect of neighbourhood interactions on final tree height irrespective of grafting (Supplementary Figure 1b). Thus, the question remains open as to whether trees are grafted because they have wider stem diameters, or they have wider stem diameters due to being grafted (Supplementary Figure 1b).

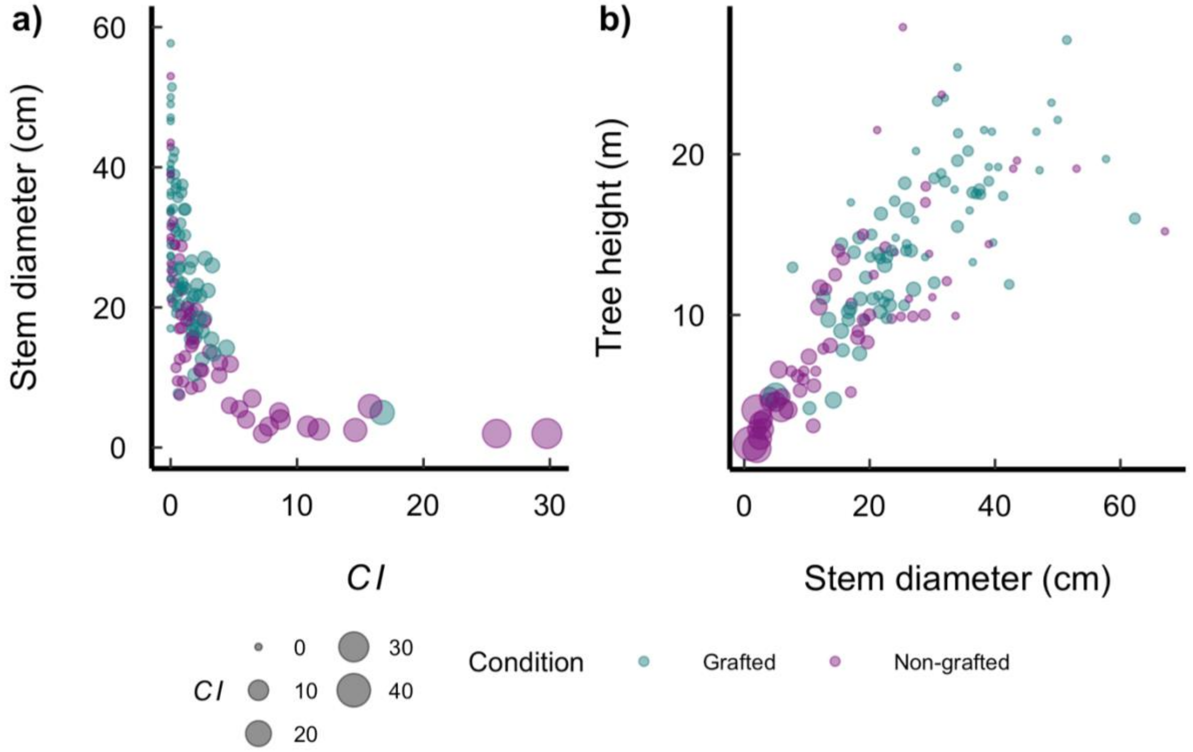

Supplementary Fig. 1: Autocorrelation of variables when using the Hegyi index. a) Strong relationship between competition index (CI) and stem diameter for grafted (blue) and non-grafted trees (purple) with increasing CI (point size) showing that higher CI values correspond to smaller stem diameters ($p < 0.001$). **b)** Relationship between tree stem diameter and height showing that the CI is reduced as stem diameters increase regardless of the grafting condition.

To deal with the autocorrelation effects of the Hegyi index, we adapted an approach to estimate asymmetric neighbourhoods as an estimate of the competitive pressure of a target $tree_i$ based on the position and size of its neighbours[5,6] defined by the following vector:

$$v = \sum_j \frac{D_{130_j}}{\left|S_i - S_j\right|^2} (S_i - S_j),$$

where v represents the vector of the neighbourhood asymmetry. The sum runs over the neighbours (j) of the target $tree_i$. D_{130_j} is the diameter of each neighbour, whereas $\left|S_i — S_j\right|^2$ denotes the squared distance between the stem position (S) of neighbour j and the stem position S of target $tree_i$, and $(S_i — S_j)$ expresses the vector between the positions of trees j and i. This approach was originally developed to understand the plastic responses of root systems to neighbours and was further applied to explore tree-crown plasticity[5–8]. It is useful for determining the relationship between neighbour sizes and grafting status as it does not assume interactions between the target tree and its neighbours. As we are only interested in the magnitude of the neighbourhood

asymmetry, not the direction, we used $|v|$ (the magnitude of vector v) and related it to each tree's stem diameter and grafting status, where for any given neighbourhood asymmetry size, there was an array of stem diameters (Supplementary Figure 2a) and an adequate exploration of tree height as a response to stem diameter and neighbourhood attributes could be assessed (Supplementary Figure 2b).

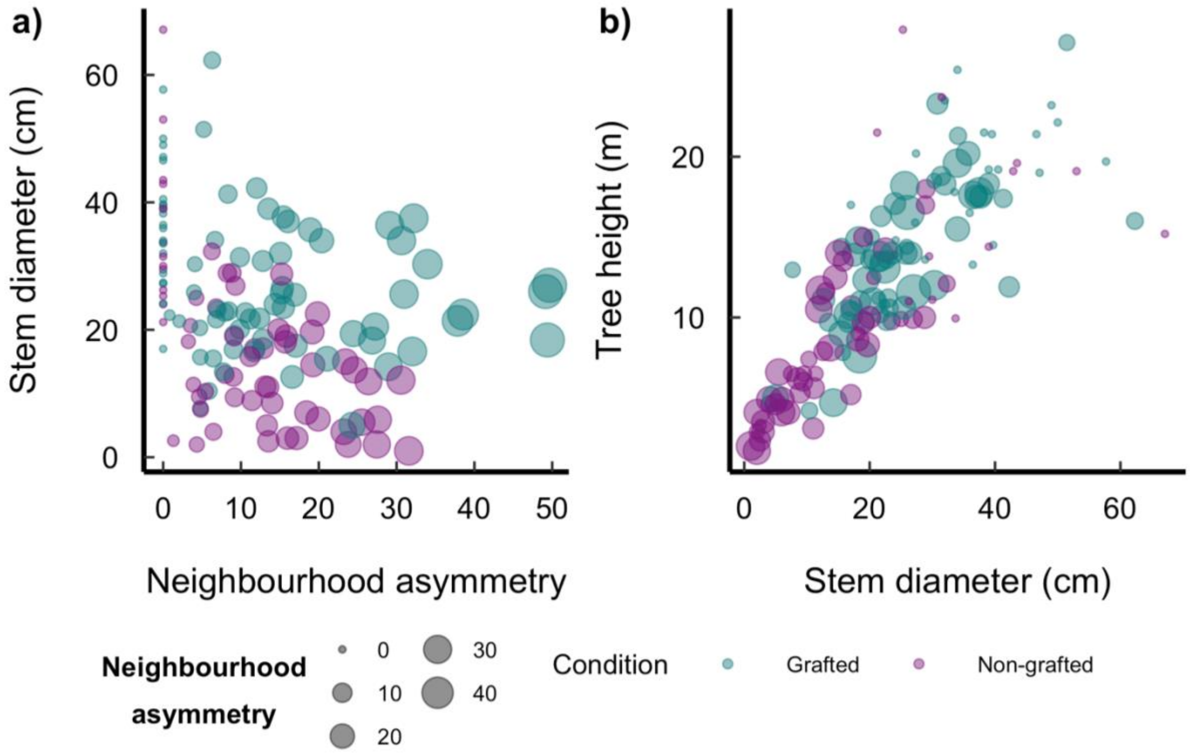

Supplementary Fig. 2: Asymmetric neighbourhoods of grafted an non-grafted trees. a) stem diameter along the magnitude of neighbourhood asymmetry (point size) showing that grafted trees (blue) have wider stem diameters than non-grafted trees (purple) regardless of the pressure of neighbours. **b)** Relationship between tree height and stem diameter of grafted and non-grafted trees showing that highly asymmetric neighbourhoods can be found over the full range of stem diameters.

As shown in Supplementary Figure 2b, a tree of any given stem diameter can have high or low asymmetrical neighbourhoods, while a large proportion of non-grafted trees belong to stem diameter classes below 15 cm.

Supplementary Table 2. Logistic regression of predictors of root grafting. Odds ratios and their 95% confidence intervals (CI) for z-transformed predictors of root grafting. Odds ratios >1 indicate a positive effect of the predictor variable on the response, while values <1 indicate a negative effect. The estimated coefficients of the odds ratios are significant if the CI range does not pass through 1. $^{+}$ denotes cross-level interactions, *indicates significant effect and · indicates a marginally significant effect.

Grafting	Odds ratio	CI 2.5–7.5
Intercept	1.45	0.99 – 2.10
Stem diameter	3.68	2.38 – 5.76*
Total density (trees ha^{-1})	0.84	0.57 – 1.22
Salinity (ppt)	0.95	0.65 – 1.37
Total density (trees ha^{-1}) : salinity (ppt)	1.10	0.75 – 1.64
Stem diameter: total density (trees ha^{-1})	0.79	0.52 – 1.17
Stem diameter : salinity (ppt)	0.58	0.37 – 0.92*
Salinity (−1 SD)	1.63	1.04 – 2.68*
Salinity (+1 SD)	0.55	0.2 – 1.07·
Total density (−1 SD)$^{+}$	1.05	0.70 – 1.67
Total density (+1 SD)$^{+}$	0.66	0.34 – 1.23

Note: Odds ratios for cross-level interactions ($^{+}$) were obtained by creating a model to specifically estimate the CI for the biggest trees in the stand (stem diameters 1 standard deviation above the mean [+1 SD]) and the smallest trees (stem diameters 1 standard deviation below the mean [−1 SD]). The estimates show an increased probability of grafting for the smaller trees in the stands with increasing salt stress, and a marginally reduced probability of grafting for the biggest trees in the stands with increasing salinity and stand density based, on 324 observations

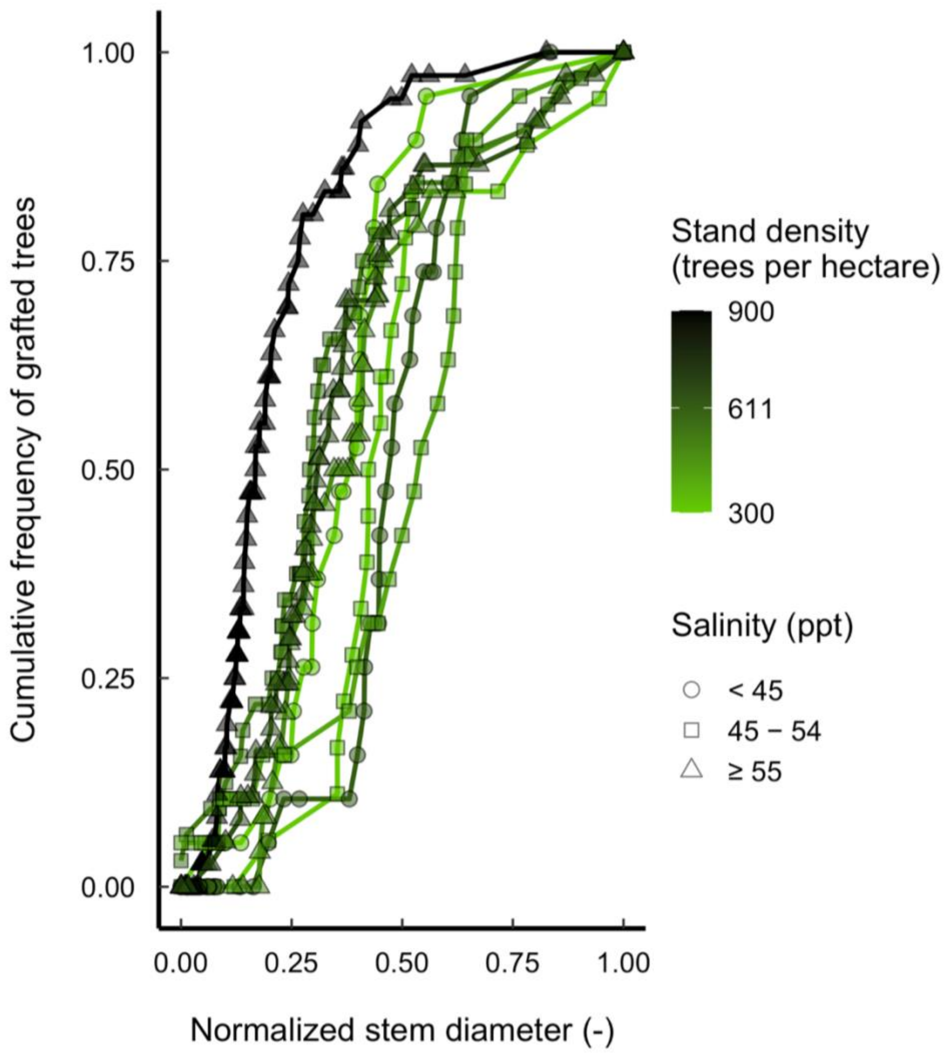

Supplementary Fig. 3: Cumulative grafting frequency on eight forest stands wit increasing stand density and sediment salinity. Increased grafting frequency is observed with increasing stem diameter (using z-transformed values for stem diameter in the model). The steepest grafting is seen for the site that had the highest salinity and stand densities.

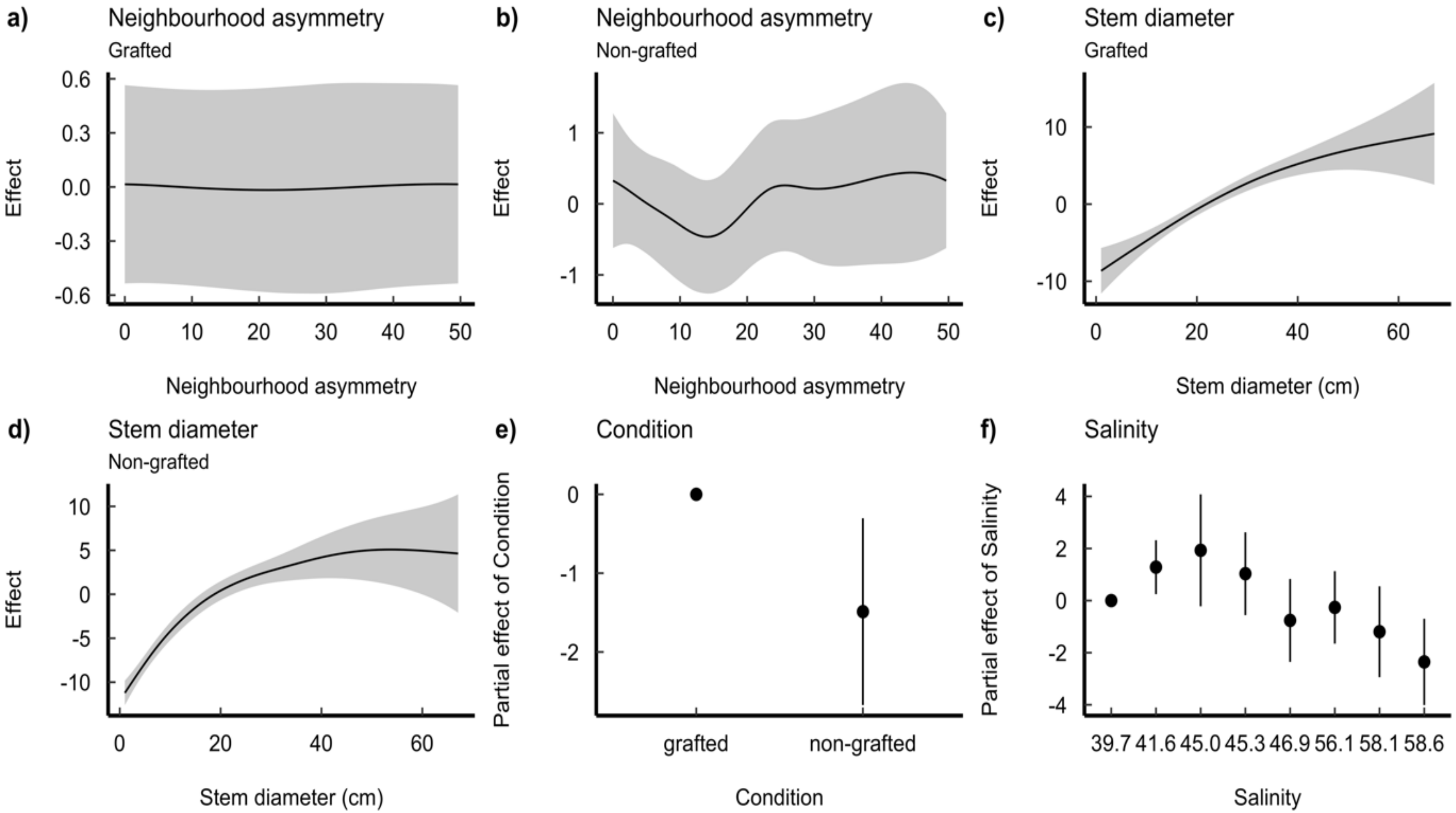

Supplementary Fig. 4: Generalized additive mixed effects model showing the effects of smooth terms on tree height. Effect of Neighbourhood asymmetry on tree height of **a)** grafted and **b)** non-grafted trees. Effect of stem diameter on tree height for **c)** grafted **d)**, non-grafted trees. **e)** effect of grafting condition and **f)** salinity on tree height, using a sample of 141 single-stem *A. germinans* trees . The shaded ribbons for panes **a**–**d** and the vertical lines in panes **e)** and **f)** express 95% confidence intervals.

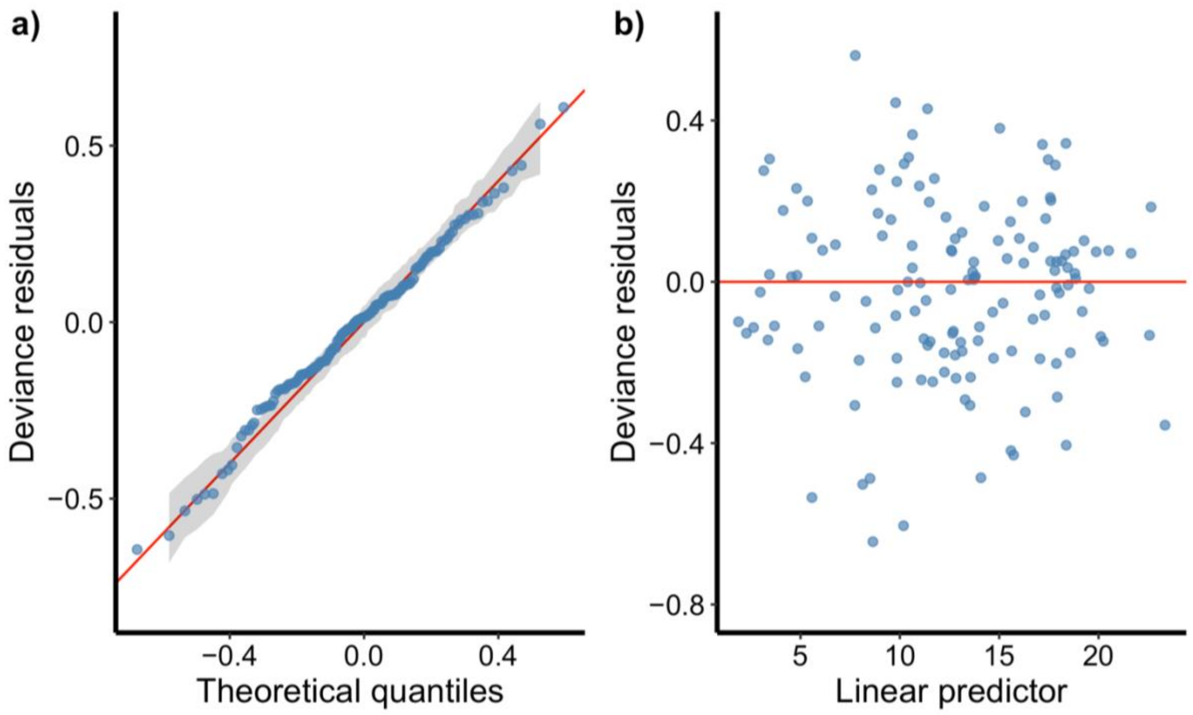

Supplementary Fig. 5: Generalized additive mixed effects model residuals. a) modelled deviance residuals vs. theoretical quantiles with 95% confidence intervals **b)** deviance residuals dispersion over the linear predictor.

Supplementary Table 3. Linear regression on slenderness and stem diameter.
NeighAsymm = asymmetric neighbourhood, NG = non-grafted condition, Sqrt(dbh) = root square transformed value of stem diameter

	**Slenderness Sqrt**		
Predictors	Estimates	CI	p
(Intercept)	11.69	9.87 – 13.51	<0.001
Sqrt(dbh)	-0.76	-1.09 – -0.44	<0.001
Condition [NG]	-0.67	-2.92 – 1.58	0.557
NeighAsymm	-0.06	-0.18 – 0.06	0.303
Sqrt(dbh) : Condition [NG]	0.05	-0.37 – 0.47	0.826
Sqrt(dbh) : NeighAsymm	0.01	-0.01 – 0.03	0.433
Condition [NG] : NeighAsymm	0.17	0.02 – 0.31	0.022
(Sqrt(dbh) : Condition[NG]) : NeighAsymm	-0.04	-0.07 – -0.01	0.010
Observations	141		
R^2 / R^2 adjusted	0.56 / 0.54		

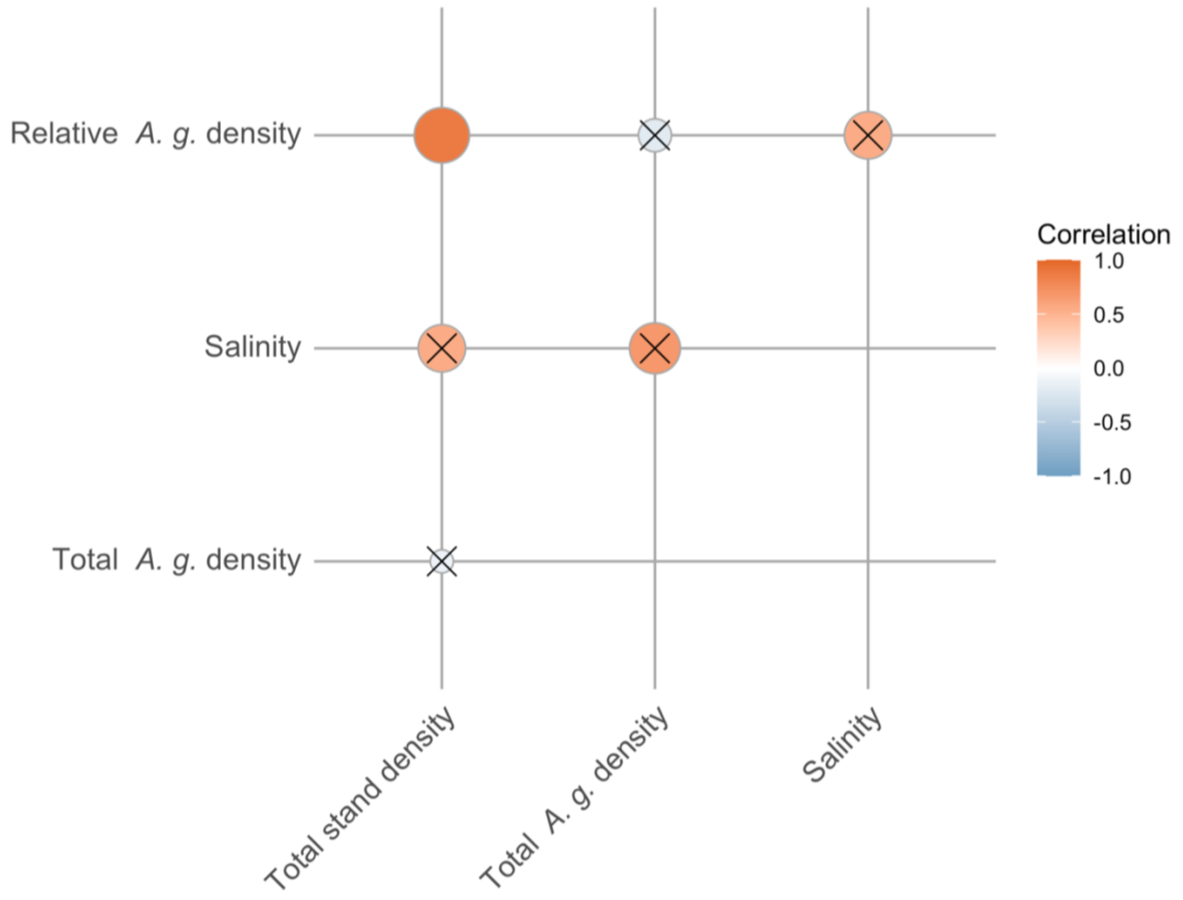

Supplementary Fig. 6: Pearson correlation of the level 2 variables in the logistic regression. X indicates non-significant correlations between variables. Relative *A. g.* density is the percentage of *A. germinans* relative to all trees (no. of *A. germinans* trees/no. total trees * 100), and Total *A. g.* density is the count of *A. germinans* trees per plot.

Supplementary References

1. Weiner, J. & Damgaard, C. Size-asymmetric competition and size-asymmetric growth in a spatially explicit zone-of-influence model of plant competition. *Ecol. Res.* **21**, 707–712 (2006).
2. Pretzsch, H. *Forest dynamics growth and yield*. *From Measurement to Model* **1**, (Springer-Verlag, 2009).
3. Berger, U., Piou, C., Schiffers, K. & Grimm, V. Competition among plants: concepts, individual-based modelling approaches, and a proposal for a future research strategy. *Perspect. Plant Ecol. Evol. Syst.* **9**, 121–135 (2008).
4. Hegyi, F. A simulation model for managing Jack-pine stands. in *Growth Models for Tree and Stand Simulation* (ed. Fries, J.) 74–90 (Royal College of Forestry, 1974).
5. Vovides, A. G. *et al.* Change in drivers of mangrove crown displacement along a salinity stress gradient. *Funct. Ecol.* **32**, 2753–2765 (2018).
6. Brisson, J. Neighborhood competition and crown asymmetry in *Acer saccharum*. *Can. J. For. Res.* **31**, 2151–2159 (2001).
7. Brisson, J. & Reynolds, J. F. The effect of neighbors on root distribution in a Creosotebush (*Larrea Tridentata*) Population. *Ecology* **75**, 1693–1702 (1994).
8. Aakala, T., Shimatani, K., Abe, T., Kubota, Y. & Kuuluvainen, T. Crown asymmetry in high latitude forests: Disentangling the directional effects of tree competition and solar radiation. *Oikos* **125**, 1035–1043 (2015).
