# Supplementary material for: Cooperative root graft networks benefit mangrove trees under stress
Source: Commun Biol. 2021 May 5;4:513. doi: 10.1038/s42003-021-02044-x (PMC8100114; doi:10.1038/s42003-021-02044-x)
Supplement: Supplementary file 4 — Reporting Summary [file 42003_2021_2044_MOESM4_ESM.pdf]

nature research

Corresponding author(s): Alejandra G. Vovides

Last updated by author(s): Mar 5, 2021

Reporting Summary

Nature Research wishes to improve the reproducibility of the work that we publish. This form provides structure for consistency and transparency in reporting. For further information on Nature Research policies, see our Editorial Policies and the Editorial Policy Checklist.

Statistics

For all statistical analyses, confirm that the following items are present in the figure legend, table legend, main text, or Methods section.

n/a	Confirmed	
☐	☒	The exact sample size (*n*) for each experimental group/condition, given as a discrete number and unit of measurement
☐	☒	A statement on whether measurements were taken from distinct samples or whether the same sample was measured repeatedly
☐	☒	The statistical test(s) used AND whether they are one- or two-sided<br>*Only common tests should be described solely by name; describe more complex techniques in the Methods section.*
☐	☒	A description of all covariates tested
☐	☒	A description of any assumptions or corrections, such as tests of normality and adjustment for multiple comparisons
☐	☒	A full description of the statistical parameters including central tendency (e.g. means) or other basic estimates (e.g. regression coefficient) AND variation (e.g. standard deviation) or associated estimates of uncertainty (e.g. confidence intervals)
☐	☒	For null hypothesis testing, the test statistic (e.g. F, t, r) with confidence intervals, effect sizes, degrees of freedom and P value noted<br>*Give P values as exact values whenever suitable.*
☒	☐	For Bayesian analysis, information on the choice of priors and Markov chain Monte Carlo settings
☐	☒	For hierarchical and complex designs, identification of the appropriate level for tests and full reporting of outcomes
☒	☐	Estimates of effect sizes (e.g. Cohen's *d*, Pearson's *r*), indicating how they were calculated

Our web collection on statistics for biologists contains articles on many of the points above.

Software and code

Policy information about availability of computer code

Data collection	No Software was used to collect data. All data was obtained from field surveys and further processed with statistical software (details in next field)
Data analysis	The R software for statistical computing (R version 3.6.2, 2019) was used to code all data analyses and figures. This includes estimates of competition, stand density, and network attributes. While all the computations described are available in the literature in some way, we have created a repository in GitHub to allow readers to access the codes if the y wish to do so. We attach a zip file with the containing GitHub repository to this application which will be made publicly available once the paper is accepted for publication.

For manuscripts utilizing custom algorithms or software that are central to the research but not yet described in published literature, software must be made available to editors and reviewers. We strongly encourage code deposition in a community repository (e.g. GitHub). See the Nature Research guidelines for submitting code & software for further information.

Data

Policy information about availability of data

All manuscripts must include a data availability statement. This statement should provide the following information, where applicable:

- Accession codes, unique identifiers, or web links for publicly available datasets
- A list of figures that have associated raw data
- A description of any restrictions on data availability

Upon acceptance of the manuscript, all data and codes used in this study will be made publicly available at https://github.com/mcwimm/GRINanalysis. We report no restrictions on data availability, just confirm that tree attributes (positions within plots, stem diameter,number of stems and height) of the study plots are already publicly available at https://doi. org/10.5525/gla.researchdata/657/

Field-specific reporting

Please select the one below that is the best fit for your research. If you are not sure, read the appropriate sections before making your selection.

☐ Life sciences ☐ Behavioural & social sciences ☒ Ecological, evolutionary & environmental sciences

For a reference copy of the document with all sections, see nature.com/documents/nr-reporting-summary-flat.pdf

Ecological, evolutionary & environmental sciences study design

All studies must disclose on these points even when the disclosure is negative.

Study description

This study presents evidence of positive interactions between trees through root grafting. We investigated that root grafting can benefit trees growing under environmental stress through the analysis of individual tree allometric traits and spatial root graft network structures. We focused, for the first time, on a mangrove forest bordering the coast of the Gulf of Mexico to understand the main drivers and consequences of natural root grafting in an A. germinans dominated forest. By relating allometric traits with presence of grafts and the magnitude of local interactions, and network topologies with stand attributes, our study shows that the forest canopy is mainly dominated by grafted trees with strong neighbourhood asymmetries (an often though measure of competition). We also demonstrate that spatial resource limitation affects network structures, limiting group sizes. These is a relevant finding that suggests the existence of underlying mechanisms involved in group-size selection, optimizing the benefit of cooperation over its costs, thus pointing to root grafting as an adaptive trait in trees that might also contribute to individual and forest resilience.

Research sample

Data was collected for a total of 482 mangrove trees found within eight 30 x 30 meter plots located along an environmental salinity gradient. From these trees, root grafts were mapped for the black mangrove (A. germinans L.) species (376 trees), out of which 52 were excluded from the logistic regression analysis to evaluate probability of grafting. The reason for excluding these trees was that they are multi-stem individuals for which the existing data base lacks information on the height of each stem. Instead it has the height of the tallest stem and it's diameter consists of the sum of all stem diameters. We consider this over estimates stem diameter and creates conflicting allometric patterns which should be addressed by taking a measurement of height for each stem.

A generalized additive mixed effect model was implemented to understand the effect of neighbourhood pressure for competition grafting condition, and physiological drought stress caused by salinity). For this neighbourhood asymmetry (an estimate of potential competitive pressure on a given tree) was calculated for all trees with a full 5m radius neighbourhood contained within the limits of the plots, and only single stem trees with their full neighbourhoods falling within the plots were used in this analysis (total of 138 trees).

Additionally a data base with a format "to-from" was constructed identifying connected trees. Form all surveyed black mangrove trees, 204 trees were found connected to at least 1 other neighbour. This data was used explore distribution functions and network attributes to understand if there are different patterns of network formation with increasing environmental stress.

Sampling strategy

No statistical methods were implemented to determine sample size. Rather, all trees contained within eight 30 x 30 m permanent plots established in the study site were identified, measured and their root connections explored with the aid of a portable doppler, a set of steel rods, and localized excavations.

Data collection

From part of an other project, since 2015, permanent plots have been established in the study site (La Mancha lagoon, veracruz mexico) along a consistent salinity stress gradient. For each plot, trees have been tagged, identified and had their attributes measured (x,y positions within plots, stem diameter, height, number of stems, and crown projection area in 8 cardinal directions). This data base is publicly available and was used to complement information gathered for this study. For each black mangrove tree, between March - May 2017, and between August -Sept 2017, within 8 of the permanent plots, root grafts were detected with the aid of a portable doppler and a set of steel rods (Vovides et al. 2016). The doppler and the steel rods aid on following woody cable roots, and when sites that indicate connections between two trees exist, localized excavations to expose the cable roots were carried out to confirm observations. All trees were examined following their cable roots from the stem towards the surrounding neighbours. Each tree was examined with this method up to where the target roots could no longer be followed due to the thinning of the roots at their distal positions form the stem.
Additionally within each plot two pore-water samples were collected form each corner and the centre of each plot and water parameters (salinity, pH, water temperature and redox potential) were determined using a Ultrameter (Myron L Company). Each pseudoreplicate was averaged and a final plot salinity concentration was estimated from the 5 samples collected

Timing and spatial scale

Tree attributes were collected for the first time in 2010, and all trees were remeasured and data bases were updated in 2015. We used the 2015 data for tree attributes. Root graft maps were recorded between March and September 2017. Water parameters used in this study were collected between April and May - September 2017

Data exclusions

52 trees were excluded from the logistic regression and GAMM analyses to evaluate probability of grafting and drivers of allmetric atributes. The reason for excluding these trees was that they are multi-stem individuals for which the existing data base lacks information on the height of each stem. Instead it has the height of the tallest stem and it's diameter consists of the sum of all stem diameters. We consider this over estimates stem diameter and could creates conflicting allometric patterns than if there was detailed information of the diameter and height of each stem within the tree.

Also trees located at the borders of the plots, for who's neighbours within a 5m radius were not fully contained within the plot, were

excluded from the GAMM analysis, as neighbours outside the plots would not have record of allometric attributes to accurately estimate neighbourhood asymmetry.

Reproducibility	Two sessions with two different students were carried out to assess the same plots and evaluate teh success in identifying root grafts using the doppler method described. While 6% of grafts had inconsistent results between students, in all cases localized excavation proved to confirm cases, and only root grafts between trees confirmed through visual inspection were labeled as grafted
Randomization	No randomization was involved in this study. As we are working along environmental gradients, it is important to ensure that environment is changing if possible only on the variable of interest. Thus, plots have been located at similar distances from the closest water cannal to warranty little variation in flooding patterns (the salinity gradient is provided by the physical location of the fresh and marine water innlet to the lagoon). Additionally, all trees are affected by their neighbours, thus all trees within plots were sampled
Blinding	No forest stands had any actively applied treatment or manipulation during data collection, thus no blinding was needed

Did the study involve field work? ☒ Yes ☐ No

Field work, collection and transport

Field conditions	The study was carried out at a coastal lagoon bordering the Central Gulf Coast of Mexico (19°33′–19°36′ N; 96°22′–96°24′ W). The annual average temperature is 25 degrees (Celcius) and is a fairly humid region with round 1,200 -1,500 mm of annual precipitation. Few hurricanes impact this coast, and the mangroves surrounding the lagoon are well preserved (300 ha of mangrove vegetation) protected since the early 1980's. Root network Field data collection took place during the year of 2017
Location	entral Coast of the Gulf of Mexico (19°33′–19°36′ N; 96°22′–96°24′ W)
Access & import/export	Permits are required by the council of wild life offices of the Enviornmental and natural resources council of Mexico (SEMARNAT) when the field work activities include the collection of biological material and/or signifficant changes to the landscape will occur. None of these apply to our study, yet we developed a particular protocol to cause the minimal disturbance possible to our study sites (see following field).
Disturbance	Disturbance on root grafting sampling was minimized by using a doppler and steel rods which dramatically minimize the need for root system excavation. Localized excavations were carried out at junctions between cable roots where suspected. These were done by hand as the sediments are soft, and covered back after visual confirmation

Reporting for specific materials, systems and methods

We require information from authors about some types of materials, experimental systems and methods used in many studies. Here, indicate whether each material, system or method listed is relevant to your study. If you are not sure if a list item applies to your research, read the appropriate section before selecting a response.

Materials & experimental systems

n/a	Involved in the study	
☒	☐	Antibodies
☒	☐	Eukaryotic cell lines
☒	☐	Palaeontology and archaeology
☒	☐	Animals and other organisms
☒	☐	Human research participants
☒	☐	Clinical data
☒	☐	Dual use research of concern

Methods

n/a	Involved in the study	
☒	☐	ChIP-seq
☒	☐	Flow cytometry
☒	☐	MRI-based neuroimaging
