# Supplementary material for: Cooperative root graft networks benefit mangrove trees under stress
Source: Commun Biol. 2021 May 5;4:513. doi: 10.1038/s42003-021-02044-x (PMC8100114; doi:10.1038/s42003-021-02044-x)
Supplement: Supplementary file 5 — Description of Additional Supplementary Files [file 42003_2021_2044_MOESM5_ESM.pdf]

Description of Additional Supplementary Files

File name: Supplementary Data

Description: Source data underlying all figures of the main text.
